# Dataset on the analytical co—hydropyrolysis of chilean oak and polyethylene under catalytic and non—catalytic conditions

**DOI:** 10.1016/j.dib.2025.112134

**Published:** 2025-10-09

**Authors:** Carlos Romero-Unda, Bastián Puentes-Navarro, Serguei Alejandro-Martín

**Affiliations:** a Laboratory of Gas Chromatography and Analytical Pyrolysis, University of Bío-Bío, Chile; b Wood Engineering Department, Faculty of Engineering, University of Bío-Bío, Chile

**Keywords:** Co-hydropyrolysis, Biomass valorization, Catalysis, Py-GC-MS, MOF derivatives, Deoxygenation

## Abstract

Co-hydropyrolysis—fast pyrolysis under a hydrogen atmosphere—offers a thermochemical route for converting plastic residues, co-processed with lignocellulosic biomass feedstocks, into platform hydrocarbons. Hydrogen-rich polymers act as in-situ hydrogen donors, while biomass-derived intermediates engage in pathways that, in the presence of bifunctional catalysts (acid sites coupled with a hydrogenating metal), promote deoxygenation, suppress polycondensation, and steer formation of BTX. A central gap is the absence of open datasets that systematically map operating conditions across matched non-catalytic (blank) and catalytic regimes within a consistent experimental framework, including raw spectra, machine-readable peak tables, and complete catalyst descriptors. Addressing this need, an open, standardized dataset is reported from a systematic study of co-hydropyrolysis of Chilean Oak with high- and low-density polyethylene in an analytical micropyrolyzer coupled to GC–MS (Py-GC–MS). Catalytic effects are quantified against catalyst-free baseline runs conducted under identical temperature, heating rate, vapour-residence time, hydrogen pressure, and feed ratio, using GC–MS relative peak areas by compound and family. Together with raw MS files, curated peak tables, operating metadata, and full catalyst descriptors (TGA, XRD, TEM, N_2_ physisorption, NH_3_-TPD; SEM–EDX available in the repository), the dataset provides an analytical basis for delineating operational windows for co-hydropyrolysis and informing process development, scale-up, and assessments of commercial viability.

Specifications Table

**Table:** 

Subject	Engineering & Materials science
Specific subject area	Thermochemical transformation of biomass and plastics into chemically relevant compounds via catalytic and non-catalytic co-hydropyrolysis.
Type of data	Tables, Figures, Raw data (TurboMass files) and Filtered data (*.xlsx files).
Data collection	Data were collected on an analytical micro-pyrolysis system (HyPy-GC–MS). Temperature, hydrogen pressure, heating rate, plastic-type, and array composition (catalyst to biomass to plastic ratios) were varied. Samples (previously conditioned) were transformed in an analytical micropyrolyzer (5200HPR, CDS Analytical, Oxford, USA). Condensable volatiles were characterized in a gas chromatograph (Clarus 690, Perkin Elmer, Cambridge, USA) equipped with a mass spectrometer (SQ-8T, Perkin Elmer, Cambridge, USA). Compound identification was performed via comparison with the National Institute of Standards and Technology (NIST, 2017, USA) database with TurboMass™ software (V5.4, Perkin Elmer).
Data source location	Institution: Universidad del Bio-Bio Affiliation: Laboratory of Gas Chromatography and Analytical Pyrolysis (LGCAP) City/Region: Concepción/VIII Región del Biobío Country: Chile
Data accessibility	Repository name: DATASET ON THE ANALYTICAL CO—HYDROPYROLYSIS OF CHILEAN OAK AND POLYETHYLENE UNDER CATALYTIC AND NON—CATALYTIC CONDITIONS. Data identification number: 10.17632/6xb48vg9rb.3 Direct URL to data: https://data.mendeley.com/datasets/6xb48vg9rb/3
Related research article	Romero-Unda C, Fernández-Andrade KJ, Vallejo F, Alejandro-Martín S. Enhanced aromatic hydrocarbon production from biomass-plastic co-hydropyrolysis over Ni/MOF-derived catalyst. Industrial Crops and Products. 2025;226:120,749. doi:10.1016/j.indcrop.2025.120749

## 1 Value of the Data

• Co-hydropyrolysis of lignocellulosic biomass and plastics-derived feedstocks emerges as a promising sustainable route to address current challenges in waste management and valorization. Consequently, analytical data are essential to delineating the operational scope of this process, particularly concerning scale-up and commercial viability.
• The data provided here enable a more detailed assessment of the distribution of chemical species within the resulting product streams and offer partial insights into the reaction pathways underpinning the complex process of catalytic co-hydropyrolysis.
• This dataset provides a valuable reference point for future studies. It can inform comparative analyses, guide experimental design, and support broader discussions on the potential of co-hydropyrolysis technologies.

## 2 Background

Global plastic wastes now exceed hundreds of millions of tonnes annually [1], imposing persistent environmental liabilities because of rapid generation, low degradability and fragmented end-of-life infrastructure. Ambitious circular-economy roadmaps increasingly position chemical recycling as a complement to mechanical routes, particularly for heterogeneous, contaminated or multi-layer streams that resist reprocessing. Within this space, catalytic thermochemical pathways—most notably pyrolysis and its hydrogen-assisted variants—offer the prospect of converting mixed residues into platform molecules and petrochemical feedstocks, thereby closing material loops under realistic quality constraints. A growing body of evidence shows that co-processing biomass with plastics unlocks valuable reaction synergies [2]. Hydrogen-rich polymers act as in-situ hydrogen donors that stabilize oxygen-centred radicals from lignocellulose, suppressing repolymerisation and coke while steering product slates towards deoxygenated hydrocarbons; under a hydrogen atmosphere (co-hydropyrolysis), deoxygenation is further enhanced and condensation pathways are curtailed. Bifunctional catalysts promote oligomerization–cyclization–aromatization cascades, enabling selective production of BTX-range aromatics under fast-pyrolysis conditions [3]. Despite these advances, the field remains hampered by heterogeneous methodologies and sparse open datasets that couple (i) full, raw files, (ii) harmonized peak tables under matched operating windows, and (iii) rigorous catalyst characterization. This limits cross-laboratory comparability, constrains kinetic/mechanistic model calibration, and slows the deployment of data-driven tools for rapid screening and process scale-up. Concurrently, MOF-derived catalysts are emerging as a versatile material to enhance acidity, metal dispersion and pore architecture, focusing on their contribution to co-conversion of biomass/plastics. However, systematic datasets resolving structure–function links across operating spaces remain scarce. The dataset presented here directly addresses these gaps by pairing Py-GC–MS raw files and curated outputs with comprehensive physicochemical descriptors for MOF-derived systems, thereby enabling reproducible benchmarking, surrogate-model training and supporting decision-making frameworks for sustainable process design. In addition, its extensive volume complements a previous critical analysis of supporting articles [2,3], providing researchers with a robust foundation to initiate or expand studies according to their specific objectives.

## 3 Data Description

### 3.1 Dataset organization

The complete dataset (*Mendeley Data*) comprises 213 files organized into four folders: (i) processed data, (ii) non-catalytic co-hydropyrolysis, (iii) catalytic co-hydropyrolysis, and (iv) characterizations. The dataset is structured as follows:

**Folder 1 – Curated data:** This folder contains two *.xlsx files compiling the processed outputs (see *Methodology for the classification of pyrolytic species* for further details) from catalytic and non-catalytic co-hydropyrolysis chromatograms. Each file consists of two or three sheets: the first provides a detailed description of the codes associated with the *.raw files, while the subsequent sheets report the relative area (R_A_, equation 1) of each compound and chemical family under the operational conditions evaluated.

**Folder 2 – Non-catalytic co-hydropyrolysis (NCHP) data:** This folder contains 81 *.raw (unprocessed) files corresponding to NCHP experiments.

**Folder 3 – Catalytic co-hydropyrolysis (CHP) data:** This folder comprises 128 *.raw (unprocessed) files corresponding to CHP experiments.

**Folder 4 – Characterizations:** This folder contains two *.xlsx files with *pristine* characterization data. The first file compiles thermogravimetric results for Chilean Oak (ChOak; *Sheet* 1), high-density polyethylene (HDPE; *Sheet* 2), ChOak/LDPE blends (*Sheets* 3–5), low-density polyethylene (LDPE; *Sheet* 6), and ChOak/HDPE blends (*Sheets* 7–9). The second file provides catalyst characterization, including TGA (Sheet 1), N_2_ physisorption (*Sheets* 2–4), XRD (*Sheet* 5), TPD-NH_3_ (*Sheet* 6), and SEM-EDX (*Sheet* 7).

### 3.2 Raw material analysis

Table 1 summarises the analytical composition of the feedstocks.

**Table 1:** Physico-chemical characterization of feedstocks.

	Biomass	LDPE	HDPE
Proximate analysis (wt % dry basis)			
Volatile matter	85.74	99.27	99.25
Fixed carbon	12.62	0.10	0.03
Ash content	1.64	0.85	0.98
HHV (MJ/kg)	20.72	45.38	44.84
Elemental analysis (wt % dry basis)			
C	47.30	85.92	86.08
H	6.36	13.67	14.00
O	46.34	–	–
N	–	–	–

### 3.3 Co-hydropyrolysis behavior

**Fig. 1:**
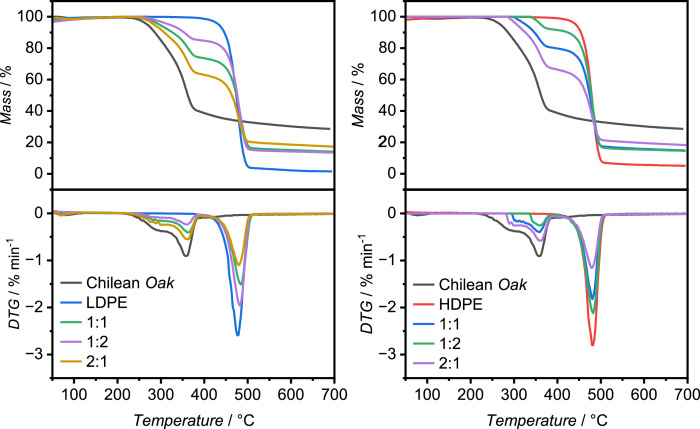
Thermogravimetric analysis (TGA) and derivative thermogravimetry (DTG) of Chilean Oak, LDPE, HDPE and their blends under hydrogen atmosphere.

Fig. 1 presents the thermogravimetric response of Chilean Oak (ChO), high-density polyethylene (HDPE), low-density polyethylene (LDPE), and their ChO/PE blends(ChO:PE = 2:1, 1:1, 1:2 by mass) under a hydrogen atmosphere, emulating co-hydropyrolysis. The mixing ratios were selected to span an oxygen-rich to hydrogen-rich envelope—bracketing biomass-dominant, stoichometrically balanced, and polymer-dominant regimes—to probe devolatilization profiles and product-slate sensitivity across practical feed compositions; the latter is evaluated in analytical co-hydropyrolysis experiments reported below. The characteristic thermal events for each polymer fraction are listed in Table 2.

**Table 2:** Non-catalytic co-hydropyrolysis thermal characterization.

	Raw materials	Maximum temperature peak ( °C)			
	Chilean Oak (ChOak)	359			
	HDPE	481			
	LDPE	476			

	Blends	T_Hemicellulose_ ( °C)	T_Celullose_ ( °C)	T_Lignin_ ( °C)	T_Plastic_ ( °C)

*ChOak*/HDPE					
	1:2	300	355	401	484
	1:1	300	359	401	480
	2:1	300	358	401	478
*ChOak*/LDPE					
	1:2	298	359	402	482
	1:1	300	361	402	484
	2:1	302	363	402	478

### 3.4 Profile of catalytic structures

Morphology and physicochemical properties were interrogated using five complementary techniques; a critical interpretation is provided in [3]. The corresponding raw datasets are supplied as an *.xlsx* workbook in Mendeley Data.

**Fig. 2:**
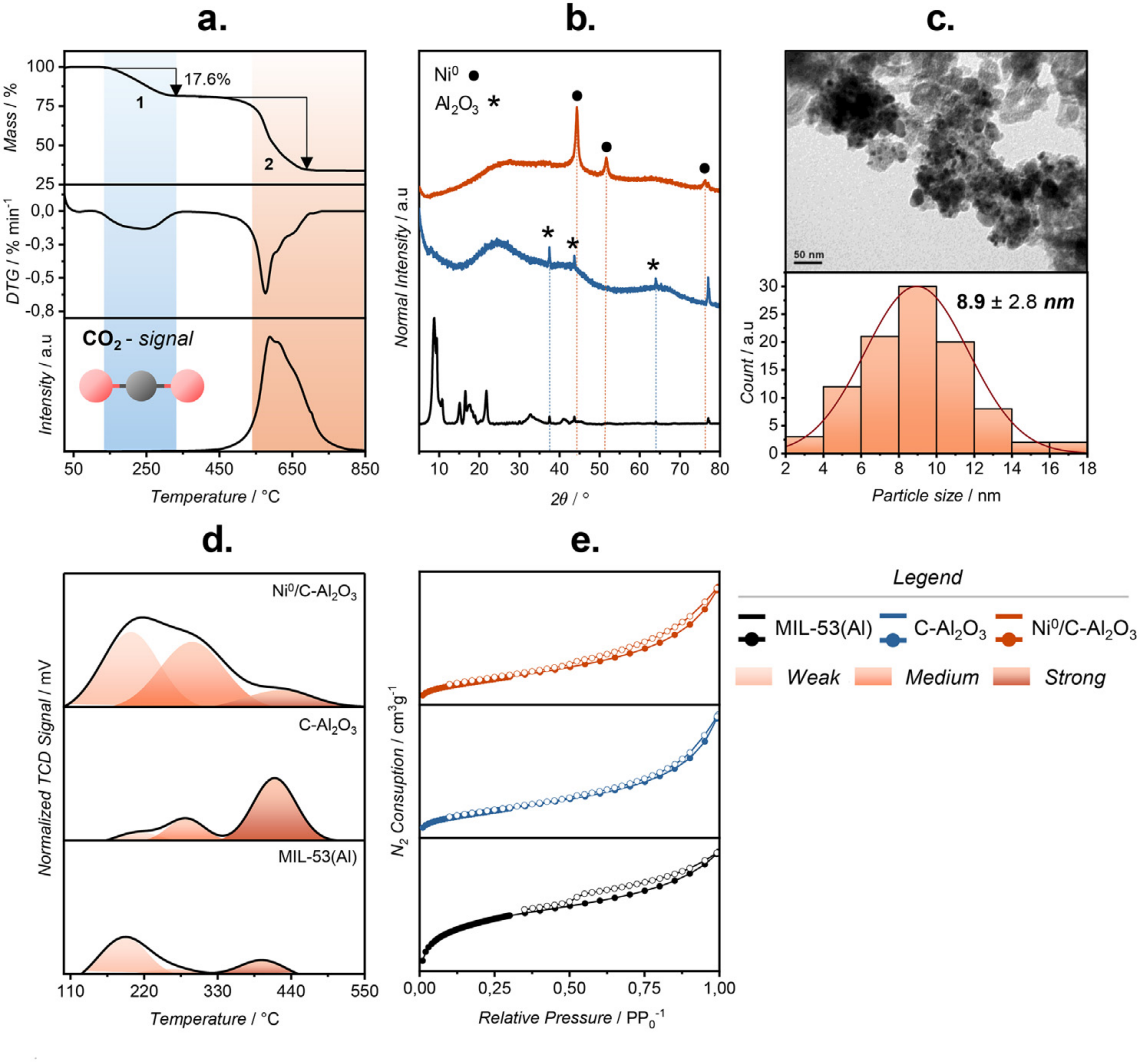
Physicochemical characterisation of MIL-53(Al), C-Al_2_O_3_, and Ni/C-Al_2_O_3_. (a) TGA-DTG profiles with CO_2_ evolution (m/z = 44). (b) XRD patterns evidencing phase and Ni incorporation. (c) TEM image and Ni particle size distribution. (d) NH_3_-TPD profiles showing acid site distribution. (e) N_2_ physisorption isotherms illustrating textural modifications after thermolysis and metal loading.

Fig. 2 compiles the characterization of MIL-53(Al), C-Al_2_O_3_ and Ni/C-Al_2_O_3_. Under N_2_ the thermoanalytical response of MIL-53(Al)—TGA/DTG coupled to mass-spectrometric detection of CO_2_ (*m/z* = 44)—shows an ∼18 % mass loss between 200 – 300 °C, attributed to solvent removal and residual precursor removal [4], followed by a ∼46 % loss at ∼570 °C associated with linker decomposition/collapse; the continuos evolution of the *m/z* = 44 signal corroborates this pathway (panel a) [5]. Powder X-ray diffraction confirms conversion from the crystalline MOF to poorly crystalline γ-Al_2_O_3_ after thermolysis (broad maxima characteristic of γ-alumina), while reflections at 2θ = 37.6°, 44.37° and 76.1° indicate fcc Ni^0^ in Ni/C-Al_2_O_3_ (panel b); partial overlap with background features near 44 – 76° is noted. Transmission electron microscopy evidences a pyrolyzed support composed of nanograins (44.3 ± 11.0 nm) decorated with well-dispersed Ni nanoparticles (8.9 ± 2.8 nm) and a narrow size distribution (panel c). Nitrogen physisorption at 77 K yields type-IV isotherms with hysteresis for all materials; relative to the parent MOF, calcination decreases the accessible surface area by ∼60 % and reorganizes the mesopore network, as reflected by BJH pore-size distributions (panel e and Table 3). NH_3_-TPD delineates the acidity landscape (panel d): MIL-53(Al) is dominated by weak sites (≈63 %); conversion to C-Al_2_O_3_ increases total acidity (∼4x) and shifts the distribution toward stronger sites (≈59 %); incorporation of Ni rebalances the population towards weak/intermediate sites (weak/intermediate ratio ≈1.2) while reducing the strong-site fraction to ≈11 % (Table 4).

**Table 3:** Textural properties of the catalysts.

Catalyst	Surface area S_BET_ (m^2^g^−1^)^a^	Pore volume V_P_ (cm^3^g^−1^)^b^	Pore size (nm)^c^
MIL-53(Al)	698	0.87	4.97
C-Al_2_O_3_	272	0.90	13.3
Ni/C-Al_2_O_3_	216	0.72	7.03

a Surface area determined through the BET model;.

b Total pore volume at P/P_0_ = 0.997;.

c Mean pore diameter (4V_P_/S_BET_).

**Table 4:** Distribution of acidic sites.

Catalyst	Acidic sites percentage			Peak *position* ( °C)		
	Weak	Medium	Strong	Weak	Medium	Strong
MIL-53 (Al)	62.66 %	23.52 %	13.82 %	195.64	272.67	398.15
C-Al_2_O_3_	10.60 %	30.44 %	58.96 %	183.74	274.34	435.2
Ni/C-Al_2_O_3_	49.46 %	40.04 %	10.50 %	220.58	312.44	431.39

### 3.5 Micro-pyrolysis assays

The experimental results provided in this dataset encompass biomass (*Nothofagus Obliqua*) and plastics (LDPE and HDPE) pyrolysis under non-catalytic and catalytic (C-Al_2_O_3_ and Ni/C-Al_2_O_3_) *reactive* atmosphere (H_2_). A specific code identifies pristine data (.raw). Table 5, Table 6 outline the main details of such identification.

**Table 5:** File title description (non-catalytic co-hydropyrolysis).

File name	
sam_bpn_p1_cohypy550_b-p_15s_1–0_100psi_oak_ldpe.raw	
Acronyms	Definition
sam_bpn	: Authors' code.
p1	: Experimental run.
cohypy550	: Co-hydropyrolysis temperature.
b-p	: Biomass-plastic.
15s	: Pyrolysis residence time.
1–0	: (Biomass)/(plastic) ratio.
100psi	: System pressure.
oak	: Biomass.
ldpe	: Plastic type.

**Table 6:** File title description (catalytic co-hydropyrolysis).

File name	
sam_cr_chp550_c2_p2_1–2_1–4_t1_hdpe_oak.raw	
Acronyms	Definition
sam_cr	: Authors' code.
chp550	: Co-hydropyrolysis temperature.
c2	: Catalyst type (c1 : C-Al_2_O_3_, c2 : Ni/C-Al_2_O_3_).
p2	: System pressure (p1 : 100 [psi], p2 : 150 [psi]).
1–2	: Biomass/plastic ratio.
1–4	: (Biomas-plastic)/(catalyst) ratio.
t1	: Heating rate (t1 : 12,5 [ °C/s], t2 : 10.000 [ °C/s]).
hdpe	: Plastic type.
oak	: Biomass.

Processed assays are detailed by compound in two .xls archives (see *Dataset Organisation* section). Rows report the operating conditions (parameters) and the corresponding R_A_ ( %). Table 7 exemplifies the processing adopted for such a purpose (see the section *Methodology for the classification of pyrolytic species* for further details).

**Table 7:** Example of catalytic co-hydropyrolysis essay report.

Family	(Biomass-plastic)/cat. ratio	1/4
	Pyrolysis press.	100
	Pyrolysis temp.	650
	Heating rate	12.5
	Biomass/plastic ratio	Oak/HDPE 1:2
	Compound / Catalyst	Ni/C-Al_2_O_3_
Acids	Oxalic acid, allyl octadecyl ester	0.51
Alcohols	1-Butanol, 3-methyl-	3.68
	1-Hexadecanol	0.27
	1-Hexanol	4.97
Esthers	Butyric acid, 2-phenyl-, dodec‑2-en-1-yl ester	0.44
	Hydratropic acid, undec‑2-en-1-yl ester	0.39
Aromatic hydrocarbons	1-Methyl-2-n-hexylbenzene	0.23
	Benzene	11.10
	Benzene, (1,3-dimethylbutyl)-	0.26
	Benzene, 1,3-dimethyl-	1.36
	Benzene, 1-ethyl-3-methyl-	0.83
	Benzene, 1-methyl-4-butyl	0.35
	Benzene, 1-methyl-4-propyl-	0.44
	Benzene, decyl-	0.24
	Benzene, hexyl-	0.40
	Benzene, n‑butyl‑	0.88
	Benzene, nonyl-	0.25
	Benzene, octyl-	0.31
	Benzene, pentyl-	0.56
	o-Xylene	0.85
	p-Xylene	1.57
	Toluene	4.35
	Naphthalene	0.71
Hydrocarbons	10-Heneicosene (c,t)	0.23
	1-Docosene	0.44
	1-Dodecyne	0.23
	1-Nonadecene	2.54
	1-Pentadecene	1.09
	1-Tetradecene	0.95
	2-Dodecene, (E)-	0.46
	2-Tridecene, (E)-	0.54
	3-Dodecene, (Z)-	0.33
	3-Undecene, (Z)-	0.75
	7-Tetradecene	0.62
	8-Heptadecene	0.24
	Cetene	0.97
	Decane	8.32
	Dodecane	4.10
	Heptacosane	1.11
	Hexadecane	2.49
	Hexane, 2-phenyl-3-propyl-	0.21
	Hexane, 3-ethyl-	3.44
	Nonadecane	9.87
	Tetradecane	6.97
	trans-3-Decene	0.86
	Undecane	4.29
Ketones	2H-Pyran-2-one, tetrahydro-6,6-dimethyl-	1.53
	1,5-Dioxaspiro[5.5]undecan-9-one, 3,3-dimethyl-	0.81
Unknown	Unknown	11.70

## 4 Experimental Design, Materials and Methods

### 4.1 Raw material

For both non-catalytic and catalytic co-hydropyrolysis, Chilean native Oak (ChO, *Nothofagus obligua*) was supplied by Miraflores Angol Ltd., Angol, Chile. The biomass was subjected to a size comminution process (sawing, chipping, grinding, and sieving) to obtain particles with diameters in the range 0.125 ≤ d_p_ ≤ 0.225 mm. Virgin high-density polyethylene (HDPE) and low-density polyethylene (LDPE) polymer beads were provided by UDT, Concepcion, Chile. These plastics were reduced in size by melting, grinding, and sieving to the same granulometry range (0.125 and 0.225 mm).

### 4.2 Biomass and plastics characterization

The properties of Chilean Oak were determined using the following standard methods: moisture content (UNE-EN 14,774), ash content (UNE-EN 14,775) and calorific value (UNE-EN 14,918). Elemental and proximate analyses were carried out according to UNE-EN 15,104 and ASTM D 3172–73(84) procedures. The elemental composition (C, H, O, N) of HDPE and LDPE was quantified using a Eurovector EA 3000 elemental analyzer.

### 4.3 Thermogravimetric analysis (TGA)

TGA experiments were performed on a NETZSCH thermobalance ST409PC (Pomerode/SC, Brazil). Approximately 10 mg of the sample was heated from 100 to 700 °C at 10 °C/min under a hydrogen flow of 20 mL/min. The sample mass was continuously recorded as a function of temperature and time.

### 4.4 Catalyst preparation

**Fig. 3:**
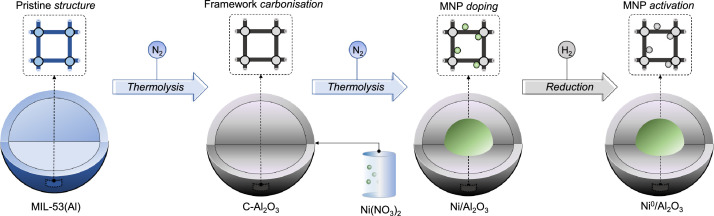
Schematic procedure for preparing catalysts.

The synthesis method and characterization of MIL-53 (Al) used as a sacrificial template for the *carbon-alumina* catalyst support have been reported previously [6]. The thermal transformation of MIL-53(Al) was achieved via high-temperature *carbonization* (slow pyrolysis): ∼200 mg of MIL-53(Al) were placed in a quartz tube (4 mm i.d.) and heated from room temperature to 800 °C at 10 °C/min for two hours under continuous nitrogen flow (100 ml/min). The resulting structure was impregnated with Ni by incipient wetness impregnation using nickel nitrate hexahydrate (Ni(NO_3_)_2_ 6H_2_O, Aldrich >98.5 %). The obtained solid (Ni/C-Al_2_O_3_) was subsequently outgassed under N_2_ flow (100 mL/min) at 800 °C for five hours, and then reduced under H_2_ flow at 550 °C (2 °C/min) for two hours to generate a catalytically active metallic nickel phase (Ni^0^). A schematic summary of the synthesis procedure is represented in Fig. 3.

### 4.5 Catalyst characterization

Thermogravimetric analysis (TGA) of MIL-53(Al) was conducted on a Netzsch-Gerätebau GmbH STA409 PC Luxx thermobalance to evaluate its thermal decomposition profile. Samples were heated from room temperature to 850 °C at a rate of 10 °C/min under a nitrogen flow of 50 mL/min. Simultaneously, a mass spectrometer monitored the evolved gases, targeting specific mass-to-charge (*m/z*) signals corresponding to H_2_O (*m/z* = 18), CH_4_ (*m/z* = 16), and CO_2_ (*m/z* = 44). X-ray diffraction (XRD) patterns of the catalysts were acquired using a Rigaku SmartLab diffractometer configured in a Theta–Theta Bragg–Brentano configuration, equipped with a D/teX Ultra 250 solid-state detector and Cu Kα radiation (λ = 1.541 Å), operated at 40 kV and 30 mA. Morphological features were examined by scanning electron microscopy (SEM) with a Hitachi SU3500 microscope, coupled to a Bruker XFlash 610 M energy-dispersive X-ray spectroscopy (EDX). Transmission electron microscopy (TEM) was carried out on a JEOL JEM-1200 EXII to further probe the internal structure of the materials. Textural characteristics were determined by N_2_ physisorption using a Micromeritics Gemini VII 2390 surface area analyzer. Prior to measurement, samples were degassed at 120 °C for 12 h under vacuum. The total acidity of the catalysts was evaluated by temperature-programmed desorption of ammonia (TPD-NH_3_). Approximately 35 mg of catalyst was pretreated by heating from room temperature to 550 °C under a continuous nitrogen flow (50 mL/min). After cooling to 125 °C, the catalyst surface was saturated with ammonia from an NH_4_OH solution. Desorption was then performed from 125 °C to 550 °C at a heating rate of 10 °C/min, with the desorbed ammonia detected by a thermal conductivity detector (TCD). To resolve the acid-strength of active sites, the TPD profiles were deconvoluted into Gaussian components using OriginPro (v9.0), spanning the characteristic temperature ranges of weak, intermediate and strong acid sites. The distribution was expressed as the percentage ratio between the area of each component and the total TPD peak area.

### 4.6 Micro-pyrolysis assays (Py-GC–MS)

#### 4.6.1 Experimental setup

**Fig. 4:**
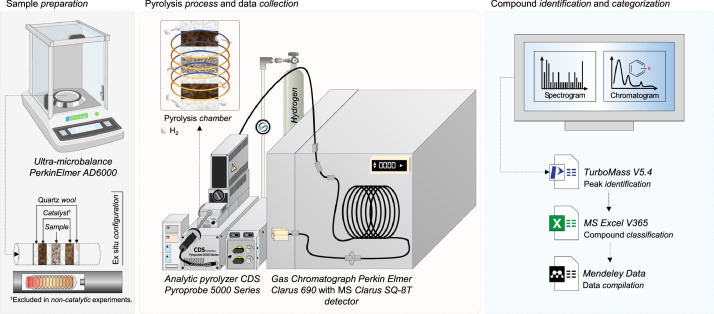
Experimental system for co-hydropyrolysis.

Chilean Oak/plastic co-hydropyrolysis was conducted in a Py-GC–MS system (Fig. 4) consisting of CDS Pyroprobe 5200HPR (CDS Analytical, Oxford, PA, USA) coupled to a Perkin Elmer Clarus 690 chromatograph (Cambridge, MA, USA), connected with a Perkin Elmer Clarus SQ-8T MS Detector (Cambridge, MA, USA).

Approximately 0.5 mg of the sample (considering ChO/plastics ratiosin Table 2 and catalyst/(ChO-plastic) ratios in Table 3) was loaded into a quartz pyrolysis tube, packed with quartz wool at both ends. The tube was placed within a Pt coil and subjected to pyrolysis under the conditions detailed in Table 8, Table 9. The evolved pyrolysis compounds were initially trapped in a Tenax trap, which was then heated to 280 °C and connected via a heated transfer line (280 °C) to the GC–MS. Compound separation was achieved using an Elite 1701 capillary column (30 *m* × 0.25 mm × 0.25 μm), operated from 45 to 280 °C with helium as carrier gas at 15 mL/min. Identification was performed by comparing the obtained mass spectra with the NIST MS library over the *m/z* range 35–300 Da. Product selectivity was quantified as relative peak area (*R_A_*), according to equation 1.

(1) $$R_{A}={A_{peak}}_{i}/\sum_{i=1}^{n}\left(A_{peak_{i}}\right)$$

where A_peaki_ is the peak area for the *i th* compound, and *n* is the total number of identified compounds.

**Table 8:** Operational parameters and experimental levels (DoE 1).

Variable	Units	Level (- 1)	Level (0)	Level (+ 1)
Plastic type	–	LDPE	–	HDPE
Pressure	psi	100	150	200
Heating rate	°C/s	12.5	25.0	10,000
Biomass content	%	33	50	67

**Table 9:** Operational parameters and experimental levels (*DoE 2*).

Variable	Units	Level -	Level +
Catalyst/(biomass-plastic) ratio	g_catalyst_/g_biomass_	4	7
Pressure	psi	100	150
Operation temperature	°C	550	650
Heating rate	°C/s	12.5	10,000
Plastic type	–	LDPE	HDPE
Biomass content	%	33	67
Catalyst type	–	C-Al_2_O_3_	Ni/C-Al_2_O_3_

#### 4.6.2 Experimental approach

The *statistical* contribution of *seven operational* parameters was examined through two distinct *Design of Experiments* (DoE). The first DoE (Table 8) investigated the non-catalytic effects of the hydrogenating atmosphere on volatile stream *deoxygenation*, while the second (Table 9) examined the impact of a *novel* catalytic structure (MOF@derived) on the selectivity towards benzene, toluene, and xylenes (BTX). The results reported in *.xls format are expressed as the *relative area* (R_A_, *equation 1*) of each compound.

### 4.7 Methodology for the classification of pyrolytic species

The chromatograms were processed using the TurboMass V6.1.0.1963 software (PerkinElmer, USA). The intrinsic parameters for signal detection (integration) [7] are presented in Table 10.

**Table 10:** Integration settings and *baseline-handling* criteria for GC–MS chromatogram processing.

Parameter	Technical specification	Graphical representation
Baselines		
Valley merging threshold	Join valleys if peaks are distinguished up to 10.00 % above the baseline. Regulates the merging level of adjacent peaks by defining the threshold at which valleys between peaks are considered part of the *same signal*.	
Trailing edge reduction	Reduce peak trailing edge elongation until the trailing edge is no >40.00 % wider than the leading edge. Adjusts peak asymmetry (geometry).	
Baseline elevation limit	Raise the baseline by no >10.00 % of the peak height. Controls interference from spurious signals (noise).	
Peak separation		
Vertical line separation	Draw a vertical line if peaks are distinguished up to 90.00 % above the baseline. Defines the criterion for separating overlapping peaks using a vertical line when their resolution reaches the specified threshold.	
Shoulder peak detection	Disabled. Allows the identification of secondary peaks if their slope is below a percentage of the maximum value.	

Compound determination was performed using a proprietary *.xlsm template. Each signal, defined by its intensity and retention time, was normalized to the total detection area (Eq. (1)), and only those with an R_A_ > 0.20 % were considered. This threshold was set as an analytical criterion to discriminate between minor signals and meaningful peaks, thereby improving reproducibility and ensuring that only compounds of quantitative significance were retained for evaluation. Chemical species were subsequently assigned by matching their fragmentation pattern with the NIST 2017 Mass Spectral Library, considering similarity indices above 700.

According to IUPAC recommendations, the identified chemical entities were categorized into eight families: alcohols, aldehydes, esters, ethers, ketones, phenols, and aliphatic and aromatic hydrocarbons (HCs). Compounds with similarity indices below 700 were classified as *Unknowns*. The total number of *Unknowns* reported in the *processed data* folder corresponds to the sum of these compounds and those with areas below 0.20 %.

## Limitations

The displayed data provides a unique (circumstantial) insight into the *organic distribution* of condensable volatiles via analytical micropyrolysis. Any experimental inference should entail a stringent adjustment of the *technical parameters* of the equipment (Py-GC–MS) and the preconditioning of the reactants.

## Ethics Statement

The dataset presented **does not** involve experiments with human subjects or animals. Moreover, it constitutes the **original data collection** performed by the research team, excluding any information from social media or other public platforms.

## Credit Author Statement

**Carlos Romero-Unda:** Conceptualization, Methodology, Investigation, Data curation, Writing – original draft. **Bastian Puentes Navarro:** Conceptualization, Methodology, Investigation, Data curation, Writing – original draft. **Serguei Alejandro-Martín:** Conceptualization, Supervision, Writing – review & editing.

## Data Availability

Mendeley DataDATASET ON THE ANALYTICAL CO-HYDROPYROLYSIS OF CHILEAN OAK AND POLYETHYLENE UNDER CATALYTIC AND NON-CATALYTIC CONDITIONS (Original data).
